# How does aging affect the types of error made in a visual short-term memory ‘object-recall’ task?

**DOI:** 10.3389/fnagi.2014.00346

**Published:** 2015-01-20

**Authors:** Raju P. Sapkota, Ian van der Linde, Shahina Pardhan

**Affiliations:** ^1^Vision and Eye Research Unit, Postgraduate Medical Institute, Anglia Ruskin UniversityCambridge, UK; ^2^Department of Computing and Technology, Anglia Ruskin UniversityCambridge, UK

**Keywords:** age differences, object-recall, memory objects, memory load, recency

## Abstract

This study examines how normal aging affects the occurrence of different types of incorrect responses in a visual short-term memory (VSTM) object-recall task. Seventeen young (Mean = 23.3 years, SD = 3.76), and 17 normally aging older (Mean = 66.5 years, SD = 6.30) adults participated. Memory stimuli comprised two or four real world objects (the memory load) presented sequentially, each for 650 ms, at random locations on a computer screen. After a 1000 ms retention interval, a test display was presented, comprising an empty box at one of the previously presented two or four memory stimulus locations. Participants were asked to report the name of the object presented at the cued location. Errors rates wherein participants reported the names of objects that had been presented in the memory display but not at the cued location (*non-target* errors) *vs*. objects that had not been presented at all in the memory display (*non-memory* errors) were compared. Significant effects of aging, memory load and target recency on error type and absolute error rates were found. Non-target error rate was higher than non-memory error rate in both age groups, indicating that VSTM may have been more often than not populated with partial traces of previously presented items. At high memory load, non-memory error rate was higher in young participants (compared to older participants) when the memory target had been presented at the earliest temporal position. However, non-target error rates exhibited a reversed trend, i.e., greater error rates were found in older participants when the memory target had been presented at the two most recent temporal positions. Data are interpreted in terms of proactive interference (earlier examined non-target items interfering with more recent items), false memories (non-memory items which have a categorical relationship to presented items, interfering with memory targets), slot and flexible resource models, and spatial coding deficits.

## INTRODUCTION

An important component of working memory model that enables recent visual events to be remembered is visual short-term memory (VSTM; Baddeley and Hitch, 1974; Baddeley, 1986, 2003). VSTM was originally proposed as the means by which recently acquired visual information is transferred into longer-term storage (Phillips, 1974). However, more recent views emphasize the importance of VSTM in the online cognition that underpins everyday tasks, such as noticing change (change detection), seeking objects (visual search), and more generally in the perception of complex scenes (Hollingworth et al., 2008).

Measurement of VSTM performance in the laboratory usually entails the brief presentation of a finite set of ‘to-be-remembered’ visual items, known as memory stimuli. After a brief retention interval, during which a blank field is typically presented, participants’ memory for the previously presented items is tested using paradigms such as change detection, yes–no recognition, or whole/partial recall (Pashler, 1988; Irwin, 1991; Irwin and Andrews, 1996; Luck and Vogel, 1997; Irwin and Zelinsky, 2002). These studies typically report that VSTM can store around 3–4 multifaceted items at any time. This apparent ceiling on the storage capacity of VSTM implies that, where more than four items are viewed, competition for storage occurs. One popular model (the *slot model*) proposes that VSTM has a fixed number of discrete *all-or-none* storage compartments (or slots); when the number of to-be-remembered items is less than the capacity of VSTM, items are remembered without a significant loss in their visual detail (Luck and Vogel, 1997; Zhang and Luck, 2008). In this model, it is believed that competition does not occur between memory items unless all slots are occupied. A more recent alternative, the *resource model*, proposes that VSTM resources are shared between the items featuring in a memory display in a more continuous fashion, such that when the number of items to be remembered exceeds the capacity of VSTM, some (correspondingly impoverished) information that pertains to a larger number of items is retained (Wilken and Ma, 2004; Bays and Husain, 2008).

Competition between items held in VSTM has been found to be influenced by several factors, including, but not limited to the items’ visual, spatial, and temporal properties. For example, items that are more familiar, visually salient and that were seen more recently in time are retained with greater accuracy than items that are less familiar, less salient (with respect to other presented items), and that were seen earlier in time (Phillips, 1983; Alvarez and Cavanagh, 2004; Zelinsky and Loschky, 2005;Hollingworth, 2007).

Another important phenomenon known to influence object representation in VSTM is our ability to inhibit information that is irrelevant (or no longer relevant) to the current task. This has been studied typically in terms of *interference* (or *intrusion*) effects (Zelinsky and Loschky, 2005; Makovski and Jiang, 2008; Fiore et al., 2012). These studies relate this interference (or intrusion) to the memory-diminishing effects arising from the items other than the memory target(s) that were also presented during the memory display (non-target memory items). In addition, interference may also originate from items retrieved from long-term memory that were not present in the memory display. These may be guesses elicited in the absence of any available memory trace, or may bear a semantic/categorical, structural, positional, or other relationship to the forgotten items that were presented in the memory display.

Error rates that arise from participant’s reporting non-target memory items for a memory target during a VSTM task have been studied extensively in young healthy participants (Irwin and Zelinsky, 2002; Zelinsky and Loschky, 2005; Makovski and Jiang, 2008; Sapkota et al., 2011). As a result of the aging population, and the commensurate increase in the prevalence of neurodegenerative conditions that affect memory performance in the elderly, research that examines age-related changes in VSTM function is becoming increasingly important. A number of studies have improved our understanding of the differences between clinically significant changes in memory function and healthy aging (De Beni and Palladino, 2004; Borella et al., 2008; Fiore et al., 2012). These studies have shown that older participants are less adept at suppressing non-target memory items compared to young participants during memory retrieval, and consequently experience greater memory distraction. The findings are compatible with the proposal that memory performance decreases with age as a result of a general deterioration in the ability of older participants to inhibit irrelevant visual information (Hasher and Zacks, 1988; Zacks and Hasher, 1994). However, previous studies that have investigated the effect of aging on VSTM performance have largely overlooked the possibility (especially in object-recall tasks) that incorrect responses may also arise due to interference from non-memory items (i.e., novel items that were not presented in the memory display). The degree to which aging may affect our ability to overcome distraction from these two types of irrelevant items during an object-recall task has not been directly compared, despite that such an investigation could have important implications for understanding the mechanisms that underpin age-related VSTM decline.

A non-target memory error can occur during an object-recall task when the binding between an item and its location has been lost, and when one of the non-target items has instead bound with the location of the memory target item. A non-memory error can occur when a memory target has been forgotten, such that interference from items that had not been presented in the memory display is greater than the interference from the items presented in the memory display. It is also possible that a well-remembered non-target memory item can be successfully excluded, leaving the participant with no choice but to guess an item that had not been presented when the memory target item has been forgotten. This may occur (for example) due to confusion between a memory target and a previously unexamined item that belongs to the same object category as the memory target (rather than report a previously examined non-target memory item, as may happen when spatiotemporal confusion between items occurs).

In this study we compared error rates in VSTM in which non-target memory items vs. non-memory items were reported during a location cued object-recall task in healthy young and normally aging older participants. In contrast to previous studies that have measured VSTM performance (e.g., capacity, longevity) wherein errors are only considered in terms of their effect on overall performance rate, a detailed analysis of the nature of error responses in this study will enable us to acquire a greater understanding of the effect of aging on the occurrence of different types of memory interference, such as proactive and retroactive interference (memory-diminishing effects arising, respectively, from stimuli examined *before* and *after* a memory target), interference from an item belonging to the same object category, and interference from other spatially nearby items (spatial proximity). To our knowledge, the influence of aging on VSTM performance during a location cued object-recall task in terms of pro/retroactive interference, spatial proximity, object category, error type, *viz*., the reporting of non-target vs. non-memory items, and its relationship with memory load and stimulus recency has not been examined before.

We predict a significant effect of age group (young or older) on the occurrence of different object-recall error types (non-target vs. non-memory), as our ability to suppress distraction arising from irrelevant (or no longer relevant) items decreases with the age (Hasher and Zacks, 1988). Furthermore, we hypothesize that older participants will exhibit greater confusion due to interference from items that are (spatially) nearby to the memory target. Also, higher error rates are expected for older participants in the high memory load condition, and for earlier presented items.

## MATERIALS AND METHODS

### PARTICIPANTS

Seventeen young (Mean = 23.3 years, SD = 3.76) and 17 normally aging older adults (Mean = 66.5 years, SD = 6.30) participated, all with normal/corrected-to-normal vision. All participants had a mini-mental state examination (MMSE) score ≥ 27, confirming normal cognitive function. Young and older participants were matched for gender (9-female, 8-male), and the minimum number of years of formal education (13 years). All participants were naïve to the purpose of the study, and were paid for their participation. Ethical clearance was obtained from Anglia Ruskin University’s Faculty Research Ethics Panel before data collection commenced. Participants were treated in accordance with applicable ethical guidelines that followed the tenets of the Helsinki Declaration.

### APPARATUS

Stimuli were displayed on a 17′′ LCD screen set at a spatial resolution of 1024 × 768 pixels and a refresh rate of 75 Hz. The screen was positioned at 57 cm from observers (such that the spatial extent of the display was ∼34° × 27°). A chin/forehead rest was used to stabilize viewing position and distance. Ambient light was held constant across trials and between participants.

### STIMULI

**FIGURE 1:**
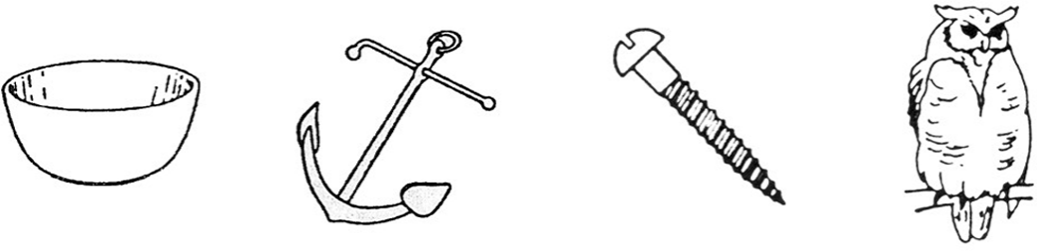
**Example stimuli**.

Stimuli comprised 170 line drawings of real world objects (Snodgrass and Vanderwart, 1980), each centered within an invisible square subtending 2.5° of visual angle at a testing distance of 57 cm. Stimuli belonged to one of 14 semantic categories (four-footed animals, birds, kitchen utensils, etc.). Example stimuli are shown in **Figure 1**. Stimulus presentation was controlled by MATLAB (Mathworks, Natick, MA, USA) with the PsychToolbox/VideoToolbox extensions (Brainard, 1997; Pelli, 1997). Stimulus background was set to mid-gray.

### PROCEDURE

Experimental procedures were preceded by a stimulus learning routine, during which all 170 stimuli were displayed sequentially in random order; participants were asked to name each stimulus as it appeared. When participants could not name/recognize a stimulus, the experimenter familiarized them with it by speaking its name aloud (i.e., a verbal prompt). Next, stimuli that participants could not name originally were re-presented one at a time, and participants were asked again to name them as they appeared. All participants were able to name all the stimuli correctly on their second attempt. Of the 13 (from 34) participants who were given verbal prompts (seven older, six young), 11 required a prompt for only one stimulus (from a total of 170), and two required a prompt for just two stimuli, indicating stimuli were nearly universally recognized, and that any priming effects arising due to stimuli being presented more than once during the learning routine were negligible. A practice block of 20 trials followed that used stimuli that were not part of the main experiment. The procedure in the practice trials was identical to that used in the main experiment (see below).

**FIGURE 2:**
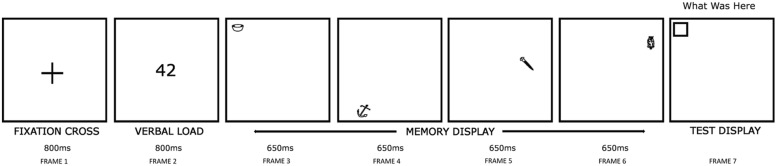
**Stimulus display procedures used (An example trial for SL4).** In the memory display (Frame 3–6), participants examined either two or four to-be-remembered stimuli at random locations. In the test display (Frame 7), an empty box appeared at one memory stimuli locations chosen at random. Participants named the memory stimulus shown at this location. In the above trial, a correct response would be ‘bowl.’

A schematic representation of the experimental procedure is provided in **Figure 2**, in which an example trial at sequence length 4 (see below) is shown. Each trial began with a 2.5° fixation cross displayed for 800 ms at the screen center (Frame 1). This ensured that all participants fixated upon a common screen position prior to the memory display. Next, a two-digit number was shown at the display screen center for 800 ms (Frame 2), followed by the presentation of a memory display (Frame 3–6), in which a sequence of either two or four stimuli [hereafter referred to as sequence length (SL)] were shown, each for 650 ms. This display duration was sufficiently long to enable stimuli to be encoded in VSTM (Vogel et al., 2006). Participants were asked to remember the object-location pairing of each stimulus (i.e., the location in the memory display at which a given stimulus was presented). Participants read aloud the two-digit number (above) whilst examining each memory stimulus to discourage verbal encoding, i.e., a *verbal suppression task* (Baddeley, 1986; Todd and Marois, 2004). Suppression tasks like this have been used previously by researchers investigating age-related changes in VSTM (Brockmole et al., 2008). Participants who were found not to be complying with the procedure were cautioned immediately by the experimenter, and the trial continued; such trials were noted down by the experimenter.

In any given trial, two or four stimuli were used in order to test memory performance at the higher and the lower end of the commonly cited 3–4 item VSTM capacity (Pashler, 1988; Luck and Vogel, 1997; Irwin and Zelinsky, 2002). Stimuli were chosen from different object categories to avoid any processing competition that may arise if the same category stimuli were used, owing to a greater number of shared properties (Bright et al., 2005). Across trials, stimuli were repeated occasionally, but with the constraint that items from different object categories were tested equally often in order to minimize ‘pop out’ effects (Jonides and Yantis, 1988). The use of a sequential (rather than simultaneous) stimulus presentation method ensured that configurational cues that could be produced by the relative spatial locations of stimuli were avoided (Jiang et al., 2000; Cowan et al., 2006; Blalock and Clegg, 2010). This isolated observed effects to VSTM specifically, and ensured that each stimulus had been fixated. In addition, a sequential display procedure ensured that any possible spatial crowding effects that may be produced by a simultaneous display were avoided, and enabled us to study the effect of stimulus recency. Each memory stimulus appeared at a new, unique screen location (chosen randomly from one of the 64 imaginary positions of an 8 × 8 square, each 2.5°); this square window, which covered the central 20° × 20° display screen area, represented the total possible area within which the memory stimuli could be displayed in any trial. Stimulus positions were never repeated within a single trial, and were at least 2.5° apart from one another. There was no delay between successive stimuli, enabling eye movements to be executed immediately after the preceding stimulus was offset (i.e., rather than drifting randomly). Moreover, the execution of eye movements between successive stimuli disrupted iconic memory that could otherwise have supported temporal integration (Eriksen and Hoffman, 1972). After a blank interval of 1000 ms, a test display was presented. This comprised a written command ‘what was here?’ and an empty square box (2.5°) at one of the randomly chosen locations used to present memory stimuli (Frame 7); participants were required to verbally report the name of the stimulus presented at that location. Where participants were entirely unsure, they were asked to report ‘I do not know.’

Participant’s (verbal) responses were recorded manually by the experimenter. The next trial started when the participant clicked a computer mouse button. The importance of accuracy (rather than speed) was emphasized to participants.

Each participant completed two blocks of 56 trials (i.e., 112 trials in total), distributed equally between sequence lengths, i.e., for each participant, for SL2, there were 56 trials in total or 28 trials per block (14 trials per temporal position per block). Similarly for SL4, there were 56 trials in total or 28 trials per block (seven trials per temporal position per block). Approximately 1 h of data capture was required per participant. Rest breaks were given between blocks; data were captured within a single session for each participant.

Performance was measured in terms of error rate, i.e., the proportion of trials in which participants incorrectly reported the name of one of the previously presented (non-target) memory items or an entirely new (non-memory) item. When non-target items were reported, the minimum 2-D Euclidean distances between each non-target item selected and the target item were calculated, and then ranked. For sequence length 4, error rates were grouped as spatial offset rank 1 (wherein the incorrectly selected non-target item was *closest* to the location of the target item), spatial offset rank 2 (where the incorrectly selected non-target item was second closest to the target item), and spatial offset rank 3 (where the incorrectly selected non-target item was *furthest* from the target item). For sequence length 2, only spatial offset rank 1 was possible.

Data were analyzed using ANOVA and *t*-tests as appropriate (see later). Where the assumption of sphericity was violated (identified using Mauchly’s test), degrees of freedom were adjusted using the Greenhouse-Geisser procedure.

## RESULTS

**FIGURE 3:**
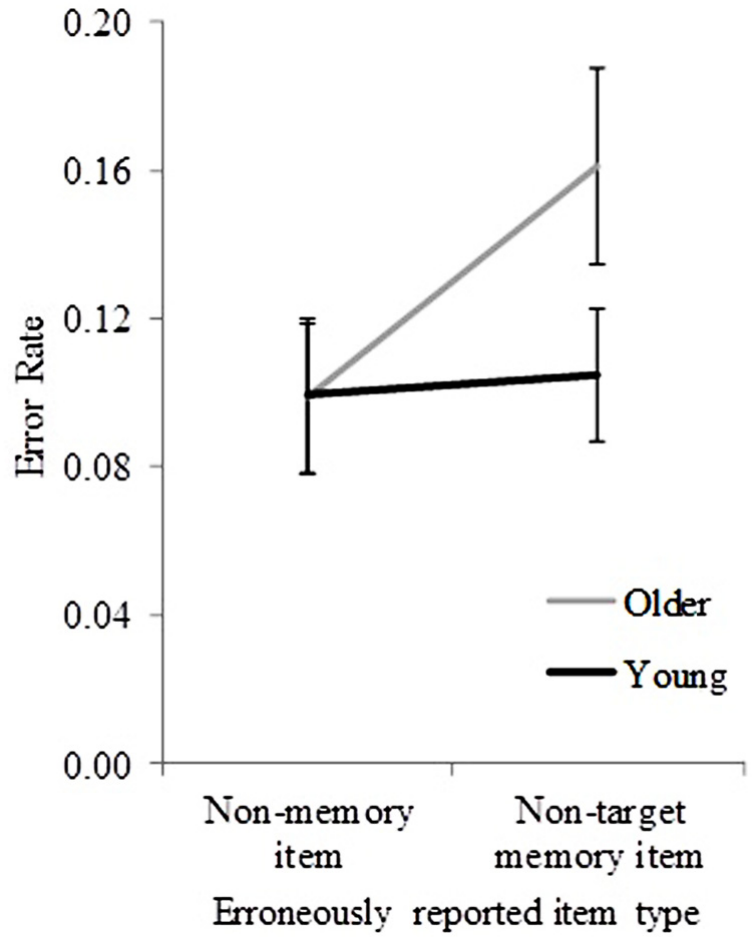
**Error rates for young and older participants (pooled across sequence lengths) for incorrectly reported non-target and non-memory items.** Error bars represent ±1.96 SE.

In less than 5% of trials, participants could not report any item at test; these trials were not subject to further analyses (except for identifying whether older participants used a more stringent criterion to choose a non-memory item, see later). In those incorrect trials in which an object name was reported at test, non-target items were reported in 56.38% of trials, and non-memory items in 43.62% of trials. **Figure 3** shows the mean error rate (pooled across sequence length) for non-target items and non-memory items for both age groups. To examine whether these pooled error rates differed significantly between age groups, a mixed ANOVA with object-recall error type (non-target and non-memory) as a within-subjects factor, and age group (young and older) as a between-subjects factor was performed. Significant main effects of age group [*F*(1,32) = 9.01, *p* = 0.005] and object-recall error type [*F*(1,32) = 7.82, *p* = 0.009] were found. A significant interaction between age group and object-recall error type was also found [*F*(1,32) = 5.37, *p* = 0.03]. The results suggest a significant effect of age group on the occurrence of different object-recall error types (non-target vs. non-memory).

**Table 1** shows mean error rates for non-target memory items and non-memory items for individual sequence length for young and older participants.

**Table 1:** Mean error rates for non-target memory items and non-memory items for individual sequence length for young and older participants.

SL	Object-recall error type	Older age group	Young age group	
2	Non-target Non-memory	0.05 (SD = 0.04) 0.07 (SD = 0.04)	0.01 (SD = 0.01) 0.06 (SD = 0.05)	
4	Non-target Non-memory	0.28 (SD = 0.09) 0.13 (SD = 0.06)	0.20 (SD = 0.07) 0.14 (SD = 0.06)	

To identify how non-target and non-memory error rates differed between age groups across memory target temporal positions for high (SL4) and low (SL2) memory loads, a mixed ANOVA was performed (seperately for non-target and non-memory items), in which temporal position (two levels for SL2, four levels for SL4) served as a within- subjects factor and age group (two levels, young and older) served as a between-subjects factor. Non-target error rates were significantly greater for older participants than young participants for both sequence lengths (**Table 2A**).

**Table 2:** **(A)** ANOVA results for comparison of error rates between young and older participants for incorrectly reported non-target memory items and non-memory items for SL2 and SL4. **(B)** *t*-test results for comparison of non-target error rates and non-memory error rates between young and older participants at each temporal position of target item presentation for SL4. (**C)** ANOVA results for comparison of error rates between incorrectly reported non-target memory items and non-memory items within each age group.

(A)				
SL	Object-recall error type	Main effect of age group	Main effect of temporal position	Interaction between age group and temporal position
2	Non-target Non-memory	*F*(1,32) = 13, *p* = 0.001 *F*(1,32) = 0.9, *p* = 0.35	*F*(1,32) = 0.90, *p* = 0.35 *F*(1,32) = 7.18, *p* = 0.01	*F*(1,32) = 2.71_,_ *p* = 0.11 *F*(1,32) = 1.0, *p* = 0.32
4	Non-target Non-memory	*F*(1,32) = 6.62, *p* = 0.01 *F*(1,32) = 0.64, *p* = 0.43	*F*(2.53,81.03) = 40.36, *p* < 0.001 *F*(3,96) = 13.05, *p* < 0.001	*F*(2.53,81.03) = 6.91, *p =* 0.001 *F*(3,96) = 3.94, *p* = 0.01

**(B)**					
**SL**	**Object-recall error type**	**Temporal position 1(young vs. older)**	**Temporal position 2(young vs. older)**	**Temporal position 3(young vs. older)**	**Temporal position 4(young vs. older)**

4 4	Non-target Non-memory	*t*(32) = -0.73, *p* = 0.47 *t*(32) = 1.92, *p* = 0.06	*t*(32) = 0.61, *p* = 0.51 *t*(32) = 1.83, *p* = 0.08	*t*(32) = -5.13, *p* < 0.001 *t*(32) = -1.30_,_ *p* = 0.20	*t*(32) = -3.57, *p* = 0.001 *t*(32) = -1.10, *p* = 0.28

**(C)**					
**SL**	**Age group**	**Main effect of error type**	**Main effect of temporal position**	**Interaction between error type and temporal position**
2	Older Young	*F*(1,16) = 2.94, *p* = 0.11 *F*(1,16) = 15.22, *p =* 0.001	*F*(1,16) = 0.12, *p* = 0.73 *F*(1,16) = 5.95, *p* = 0.03	*F*(1,16) = 3.39, *p* = 0.84 *F*(1,16) = 2.48, *p* = 0.13
4	Older Young	*F*(1,16) = 26.02, *p* < 0.001 *F*(1,16) = 5.11, *p* = 0.04	*F*(3,48) = 25.75, *p* < 0.001 *F*(3,48) = 50.04, *p* < 0.001	*F*(3,48) = 5.14, *p =* 0.004 *F*(3,48) = 3.72, *p* = 0.02

A significant interaction between age group and memory target temporal position was found for SL4 but not SL2 (**Table 2A**). This suggests that interference from non-target memory items at high memory load affected each age group differently, depending upon whether the memory target was probed at earlier or more recent temporal positions. Non-memory error rates, however, did not differ significantly between age groups for either sequence length, although a significant interaction between age group and memory target temporal position was found for SL4 (**Table 2A**), suggesting that interference from non-memory items at high memory load also affected each age group differently, depending upon whether the memory target was probed at earlier or more recent temporal positions. Furthermore, these results suggest that older participants incurred greater intrusion (compared to young participants) from non-target memory (but not from non-memory) items at both high and low memory loads.

**FIGURE 4:**
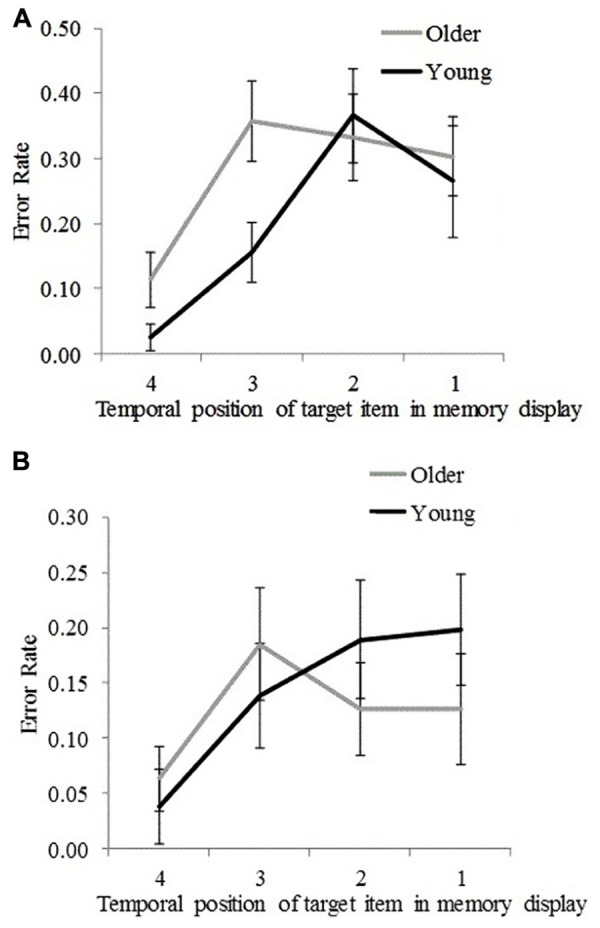
**Error rates for young and older participants for each temporal position (1 = earliest, 4 = latest) at which the target stimulus was presented in the memory display. (A)** For incorrectly reported non-target memory items. **(B)** For incorrectly reported non-memory items. Error bars represent ±1.96 SE.

Plotting error rate as a function of the memory target temporal position enables us to visualize the significant interaction effects (reported above) between age group and memory target temporal position for non-target and non-memory items for SL4 (**Figures 4A,B**).

To determine at which temporal positions non-target error rates differed significantly between the two age groups, an independent samples *t*-test was performed. Results are presented in **Table 2B**. Significantly greater non-target error rates were found for older participants compared to young participants for the third and the fourth temporal positions but not for the first and the second temporal positions (**Table 2B**). This suggests that older participants incurred greater proactive interference compared to young participants. An inverse trend was observed for non-memory error rates (*cf*. **Figures 4A,B**), in which younger participants showed greater (nearing the significance threshold of 0.05) error rates compared to older participants when the memory target was presented at the earliest two temporal positions (**Table 2B**). This effect may have arisen because older participants used a more stringent criterion for a non-memory item response when the memory target was presented at the first and second temporal positions. This assertion is supported by an analysis of the frequency of those trials in which participants could not report any item at test (although these occurred in less than 5% of all trials), which were more common for older participants than young participants, *t*(32) = 2.76, *p* = 0.009.

To examine temporal position effects in SL4 trials, a one-way repeated measures ANOVA was performed on combined error rate data (non-target and non-memory), using memory target temporal position (four levels for SL4) as a within-subjects factor. A significant temporal position effect was found for both age groups [older participants, *F*(3,48) = 26.13, *p* < 0.001; young participants, *F*(3,48) = 50.36, *p* < 0.001], suggesting that more recent items were remembered with greater accuracy (i.e., global recency effects).

To examine how object-recall error types differed from each other *within* each age group across memory target temporal positions, separate analyses were performed for SL2 and SL4 as follows: 2 (error type, non-memory and non-target) × 2 (temporal position) repeated measures ANOVA for SL2; and 2 (error type, non-memory and non-target) × 4 (temporal position) repeated measures ANOVA for SL4. Results are presented in **Table 2C**. At high memory load (SL4), non-target error rates were significantly greater than non-memory error rates for both age groups. A significant interaction between object-recall error type and memory target temporal position was found. This suggests that, for both age groups, at high memory load, object-recall error types varied differently as a function of memory target recency. At low memory load (SL2), a reverse trend (non-memory error rates greater than non-target error rates) was observed, although the difference attained statistical significance only for young participants (**Table 2C**). No significant interaction between the object-recall error type (non-target and non-memory) and memory target temporal position was found for SL2. These results suggest that, as memory load increases from 2 to 4 items, greater interference occurs from non-target memory items when compared to non-memory items in both age groups.

To examine whether the incorrectly reported non-target memory items were more likely to be spatially proximal to the memory target (spatial proximity), a one-way repeated measures ANOVA was performed, separately for each age group, on non-target error rates for SL4 using spatial offset (of the non-target item reported relative to the memory target) ranks 1–3 as a within-subjects factor. A significant main effect of spatial offset rank was found in both young [*F*(2,32) = 4.30, *p* = 0.02] and older [*F*(2,32) = 16.63, *p* < 0.001] participants. To establish, specifically, at which spatial offset ranks non-target error rates differed significantly between age groups, an independent samples *t*-test was performed on error rates at each spatial offset rank of 1–3 between young and older participants. Older participants were found to exhibit greater error rates at spatial offset rank 1 [*t*(32) = 2.60, *p* = 0.01], and rank 2 [*t*(32) = 3.76, *p* = 0.01] compared to younger participants. No significant difference was found at spatial offset rank 3 [*t*(32) = 1.20, *p* = 0.24] between the two age groups. This suggests that age-related differences in VSTM error rate are influenced significantly by non-target items presented at nearby locations (albeit sequentially) compared to those that are more distant. The average calculated distance between a memory target and the non-target item reported was found to be less than the average distance between all non-target items presented in SL4 trials [older participants, 10.99 vs. 12.06°; young participant, 11.55 vs. 11.95°]. To identify whether these distances differed significantly from one another within each age group, a paired samples *t*-test was performed separately for young and older participants. A significant difference in older [*t*(16) = 2.56, *p* = 0.02], but not in young participants [*t*(16) = 1.29, *p* = 0.21] was found, suggesting that older participants are more likely to select non-target items that are closer to the memory target. This is further supported by a mixed ANOVA performed on absolute distances (i.e., between the memory target and erroneously reported non-target items) using spatial offset ranks (1–3) as a within-subjects factor, and age group (young and older) as a between-subjects factor, in which a significant main effect of age group was also found [*F*(1,32) = 4.49, *p* = 0.04].

One may suggest that non-memory items presented in earlier trials at the location where a memory target is presented in subsequent trial may intrude significantly upon the memory target item. However, errors for non-memory items that had been presented during earlier trials at the location where a memory target was subsequently presented occurred in less than 1% of trials in both age groups, suggesting that non-significant intrusion occurred from the items presented in earlier trials at the location of a memory target item.

**Table 3** shows the distribution of errors depending upon whether incorrectly reported non-memory items belonged to the same or a different object category relative to the memory target. In 25% of trials for older participants, and 28% of trials for young participants, an item that was not examined in the memory display, but belonged to the same object category as the memory target was reported, suggesting that category information was retained across trials even if detailed item information may have been lost. However, there was no significant difference between the two age groups [*t*(32) = 0.84, *p* = 0.40], demonstrating that VSTM performance does not differ significantly between age groups as a consequence of competition from unseen items belonging to the same object category as the memory target.

**Table 3:** Non-memory errors (pooled across sequence lengths) for young and older participants depending upon whether the incorrectly recalled item belonged to the same or different object category to the memory target.

Incorrectly recalled object category	Older participants	Young participants
Different to memory target	75% (SD = 13%)	72% (SD = 9%)
Same to memory target	25% (SD = 13%)	28% (SD = 9%)

## DISCUSSION

In this study, we investigated how different types of VSTM errors (i.e., the incorrect reporting of non-target memory and non-memory items) in a location-cued object-recall task differed within and across age groups. We examined how these differences varied with memory load (the number of items to be remembered) and target stimulus recency. Overall, a greater error rate for non-target memory items compared to non-memory items was found in both young and older participants; however, older participants performed less well overall. At the high memory load (SL4), non-target error rates occurred more often than non-memory error rate in both age groups. However, at the low memory load (SL2), non-memory error rates occurred more often than non-target error rates, although the difference attained statistical significance only in young participants. Furthermore, at the high memory load, young participants exhibited greater error rates (compared to older participants) for non-memory items when the memory target was presented at the earliest two temporal positions. This is possibly due to older participants using a more stringent criterion for producing a non-memory item response. However, a reverse trend was observed for non-target error rates, which were significantly greater for older participants compared to young participants when the memory target was presented at the two most recent temporal positions (**Figures 4A,B**), suggesting that older participants incurred more proactive interference compared to young participants (Bowles and Salthouse, 2006). Furthermore, non-target error rates (in both age groups) were found to asymptote at the earliest (first and second) temporal positions, countering the predictions of the slot-based model of working memory. According to this model, a fixed number of slots are assumed to exist in VSTM, such that with each newly examined item the probability of forgetting an earlier examined item increases for high memory load. If it was the case that lower memory performance (specifically, greater non-target error rate) for older participants was a consequence of their having fewer slots, the probability that earlier items would be forgotten would be greater (compared to young participants) in SL4 trials. This was not found with our data. The finding that young participants exhibited reduced non-target error rates compared to older participants when the memory target item was presented at the two most recent temporal positions rather suggests that the memory resources allocated for more recently examined items are prioritized better by young participants above less recently examined items, potentially as a result of greater encoding fidelity, attention, resource redeployment to favor more recent items, or superior executive control. This assumption is compatible with the flexible resource model (Wilken and Ma, 2004; Bays and Husain, 2008).

The finding of a greater error rate overall for older participants may be explained by inhibitory deficits, i.e., the ability to inhibit non-target memory items decreases with age (De Beni and Palladino, 2004; Bowles and Salthouse, 2006; Fiore et al., 2012). In addition, our findings provide evidence for the following factors that influence object representation in VSTM: (i) at high memory loads we incur greater interference from non-target memory items, and at low memory loads we incur greater interference from non-memory items; (ii) we are less adept at remembering less recent non-target memory items, as a consequence of interference incurred from subsequent items (retroactive interference).

One may argue that our findings could have been confounded by systematic differences between young and older participants’ gaze control (i.e., shifting the gaze from one stimulus in the sequence to the next). It should be noted that our stimuli were presented one at a time with a display duration that was sufficiently long (at 650 ms) for each object to be captured by the visual system, and gaze to be shifted to the subsequent object. Although we did not record the eye movements directly, we noted those trials in which participants either forgot to read aloud the verbal load (which they were supposed to read aloud every time a memory stimulus was presented), or read it aloud an incorrect number of times (i.e., differing from the number of stimuli presented in the memory display). Such errors were found to occur in less than 2% of trials. Furthermore, no significant differences were found in these trials between young and older participants, suggesting that our results were not confounded by systematic age-related differences in eye-gaze control.

A number of hypotheses are offered as to why VSTM performance may decline with advancing age. Salthouse (1990, 1994) suggests a generalized slowing of overall cognitive processes, while Craik and Byrd (1982) argue for a progressive deterioration in available attentional resources. Others propose impoverished memory representations owing to lower-fidelity sensory inputs (Rabbitt, 1991; Lindenberger and Baltes, 1994; Baltes and Lindenberger, 1997; Murphy et al., 2000), or a general deterioration in our ability to inhibit visual information belonging to objects that are irrelevant to our current goals (Hasher and Zacks, 1988; Zacks and Hasher, 1994). Yet another hypothesis proposes that VSTM performance declines with advancing age due to a decreased ability of older participants to encode and retrieve associations between constituent object features stored in VSTM (Chalfonte and Johnson, 1996; Mitchell et al., 2000; Naveh-Benjamin, 2000; Cowan et al., 2006). While the aim of this research was not to test one or the other of these hypotheses, our finding of significant age-related differences in proactive interference is better explained by inhibitory deficit hypothesis (Hasher and Zacks, 1988; Zacks and Hasher, 1994). Our findings add to the inhibitory deficit hypothesis by proposing that age-related VSTM decline is influenced by interference originating from items examined *earlier* than a memory target (proactive interference), but not from items examined *after* a memory target (retroactive interference). Similarly, age-related decline in VSTM may also occur due to interference from non-target items that are examined spatially nearby to the memory target, suggesting that spatial coding deficits become more pronounced in VSTM with advancing age.

To summarize, our data demonstrate that normal aging affects VSTM performance in an object-recall task generally, and more specifically that these aging effects were modulated by memory load and target stimulus recency. Overall error rates differed significantly between age groups when the incorrectly reported item was one of the previously presented non-target items, but not when an entirely new item (non-memory item) was reported. For both young and older participants, at the high memory load, error rates for non-target memory items were greater, whereas at low memory load, error rates for non-memory items were greater. Older participants showed higher non-target error rates when the memory target was presented at the two most recent temporal positions (but not at earlier temporal positions). This suggests that proactive interference was greater for older participants compared to young participants. Similarly, greater interference from non-target items that were spatially nearer to the memory target was found for older participants compared to young participants, suggesting impaired spatial coding for older participants. Future studies might consider the influence of changes in other variables, such as stimulus duration, retention interval, and response time, in order to compare age-related differences in the memory decline associated with non-target memory items and non-memory items at each stage of VSTM processing, *viz*. encoding, maintenance, and retrieval.

Our findings have important implications for understanding the mechanisms that underpin age-related VSTM performance decline, and suggest that a less cluttered visual environment may be particularly beneficial to the elderly by reducing the number of irrelevant visual items (that they are less adept at inhibiting), which may improve their performance in everyday visual tasks requiring VSTM. Furthermore, our results are relevant to ongoing debate concerning the most appropriate working memory model, such as the resource (Wilken and Ma, 2004; Bays and Husain, 2008) and slot models (Zhang and Luck, 2008). A lower non-target error rate for young participants compared to older participants was found when the memory target was presented at the two most recent temporal positions in SL4. This may suggest that young participants are more efficient at redeploying memory resources allocated to earlier presented items for more recent items, exhibit greater attention or executive control, or dynamically prioritize new stimuli over earlier stimuli, an assumption that is compatible with the flexible resource model (Wilken and Ma, 2004; Bays and Husain, 2008). Furthermore, our results suggest that VSTM resources are not only shared between recently examined items, but also with items that were examined in the distant past (i.e., from our prior visual experiences), which can produce false memories.

## Conflict of Interest Statement

The authors declare that the research was conducted in the absence of any commercial or financial relationships that could be construed as a potential conflict of interest.
